# Comparison of dislocation rates of Teflon and Titanium stapes prostheses: a retrospective survival analysis on 855 patients

**DOI:** 10.1186/s40463-023-00654-5

**Published:** 2023-08-11

**Authors:** Stéphane Gargula, Mary Daval, Adrien Lecoeuvre, Denis Ayache

**Affiliations:** 1 Department of Otolaryngology, Hôpital Fondation Adolphe de Rothschild, 29 Rue Manin, 75019 Paris, France; 2 Clinical Research Unit, Hôpital Fondation Adolphe de Rothschild, 29 Rue Manin, 75019 Paris, France

**Keywords:** Stapes, Otosclerosis, Stapes surgery, Titanium, Incus, Survival analysis

## Abstract

**Background:**

Stapes prosthesis dislocation is the first cause of revision stapes surgery. To our knowledge, there is no data about stability of the incus attachment of manual crimped prosthesis of different materials. This study aimed to compare the dislocation incidence between titanium and fluoroplastic stapes prostheses.

**Method:**

A monocentric retrospective cohort study was conducted between January 2013 and June 2022 in a tertiary-care center. All patients that underwent a primary stapes surgery with manually crimped fluoroplastic or titanium prostheses were included. Prosthesis dislocation from the incus was identified intraoperatively or with CT scan. The incidence of stapedial prosthesis dislocation over time was estimated using the Kalbfleisch and Prentice survival analysis method. Other indications for revision surgery prior to prosthesis dislocation were considered as competing events. Differences in the cumulative incidence functions between the fluoroplastic group and the titanium group was assessed using the Gray’s test.

**Results:**

Eight hundred and fifty-five patients underwent primary stapes surgery during the study period. Fluoroplastic prosthesis was used in 758 (88.7%) cases and titanium prosthesis in 97 (11.3%) cases. Median follow-up was 51.7 months (28.4–80.1). Dislocation was observed in 23 (3.0%) patients with fluoroplastic prosthesis and none (0.0%) in the titanium group. The probability of prosthesis dislocation at two years after surgery was 3.5% in the Teflon group and 0.0% in the Titanium group. No significant difference was found in the cumulative incidence of prosthesis dislocation between the fluoroplastic group and the titanium group (p = 0.12).

**Conclusions:**

Despite lack of statistical power, our results suggest a trend in a more stable incus attachment of manually crimped titanium stapes prosthesis compared to fluoroplastic over time. Further prospective randomized studies could be valuable to assess our findings.

**Graphical abstract:**

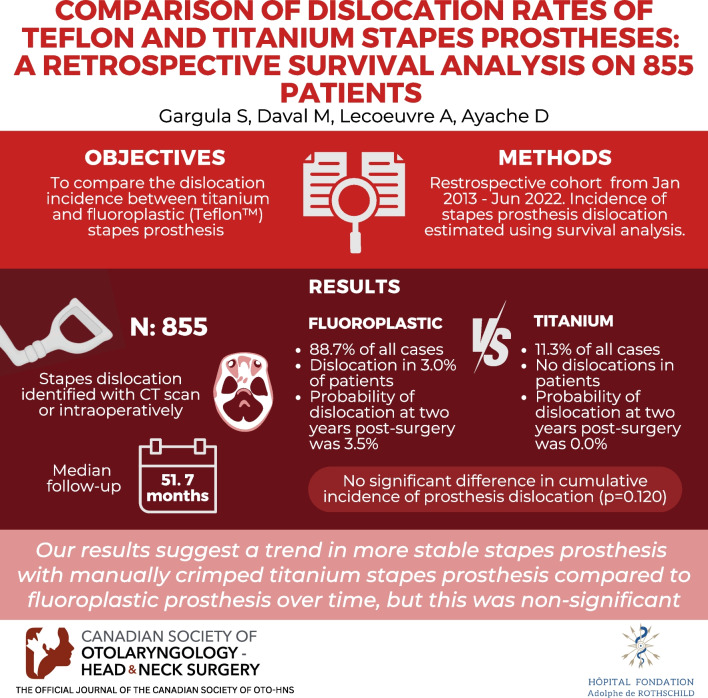

## Background

Otosclerosis is a common cause of conductive or mixed hearing-loss (CMHL) [1]. Stapes surgery is a popular treatment modality, with high success rates [2–5]. Various types of stapes prosthesis have been developed, but comparison of the hearing results of different materials and crimping methods have failed to demonstrate the superiority of particular prosthesis. Fluoroplastic (Teflon) and Titanium manually crimped prosthesis are amongst the most used, and have comparable hearing results [6, 7].

As it affects young adults, the stability of the prosthesis over time is a major concern. Dislocation of the prosthesis is the first cause of recurrence of hearing loss, leading to revision surgery, which has a lower success rate [8–10]. However, there is very little data of prosthesis dislocation rate after stapes surgery, even in long term studies [5, 11]. To our knowledge, there is no study in the literature comparing the risk of dislocation regarding the material used.

The aim of our study was to compare the dislocation rate of Teflon and Titanium prosthesis.

## Methods

This is a retrospective cohort study performed in an otolaryngology tertiary-care center, covering a period between January 2013 and June 2022. This retrospective study has been approved by the institutional review board “IRB 00012801” under the validation number ID “CE_20220726_4_OTOPIST”. The STROBE guidelines were used for reporting [12].

### Population

All patients who underwent primary stapedotomy for otosclerosis between January 2013 and June 2022 were included. Eligible patients were identified from two sources, the surgical procedure register and the traceability register of implanted prostheses. If the patient had surgery on both sides, only the first operated side was considered (as survival analysis depends on independence of events).

Data were collected from hospital medical records. The following variables were collected: age, sex, prosthesis used, the delay and the motive for revision surgery if applicable, with detailed intraoperative findings.

The 4.5 × 0.6 mm Teflon piston is the standard prosthesis in our centre for stapes surgery. A titanium (BigEasy, Medtronic) 4.5 × 0.5 mm piston is used in case of selected intraoperative presentation: smaller shaft and foot of prosthesis needed (narrow oval window, facial canal or nerve bulging), surgeon subjective feeling for need of tighter crimping on the long-process (LPI) of incus (frail LPI, erosion of the lenticular process). The surgery is performed through endaural procedure with intercartilaginous incision. Stapes footplate fenestration is performed with a calibrated manual perforator, with or without assistance of a diode fibre laser. Facial canal dehiscence is a contraindication for laser use. If needed, connective tissue is used to prevent leakage around the shaft, or fascia interposition to seal the oval window niche when the footplate is damaged.

### Study outcomes

**Fig. 1:**
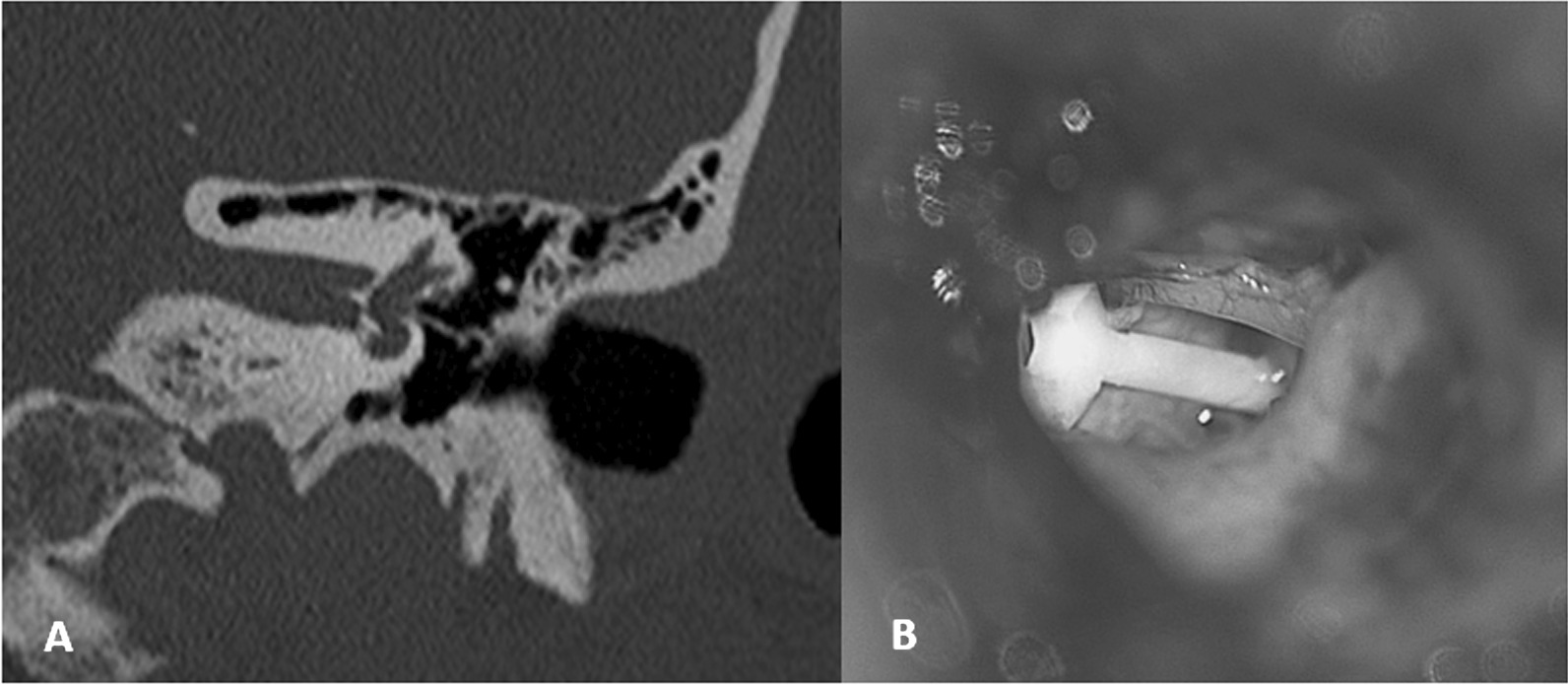
**A** Left ear CT-scan with coronal reconstruction in the axis of a dislocated prosthesis. **B** Per-operative view of a dislocated piston during revision stapes surgery of a left ear

The primary endpoint was the occurrence of stapedial prosthesis loop dislocation from the LPI during follow-up. Diagnosis of prosthesis dislocation was based on intraoperative findings during revision surgery, or on temporal bone CT scan with multiplanar reconstruction analysed by both a neuroradiologist and an otologist (Fig. 1) [13]*.*

To avoid bias, the following situations lead to exclusion of the patients from the analysis: use of cement during the primary surgery, use of a different type of prosthesis (i.e., malleostapedotomy prosthesis).

The secondary outcomes were the occurrence of other post-operative events (sensorineural hearing loss and other causes of CMHL), and intraoperative findings of revision surgery for prosthesis dislocation. The records of dislocation cases were carefully reviewed for descriptive analysis of the intraoperative presentation. For descriptive reports LPI was considered mildly eroded when only the lenticular process was eroded allowing a conventional procedure, moderately eroded when it compromised the attachment of the piston loop requiring cement stabilisation, and severely eroded when the incus could not be used and malleostapedotomy was performed.

### Statistical analysis

Qualitative variables were described as the frequency (percentage). Normally distributed quantitative variables were described as the mean (SD) and non-normally distributed quantitative variables were described as the median [IQR]. The distribution was assessed using histograms and Q-Q plots.

Patient baseline fluoroplastic and titanium group characteristics were compared using Pearson’s Chi-squared test or Student t-tests, as appropriate, for each variable.

As subjects can experience early surgical revision for postoperative SNHL and other causes of revision surgery for recurrence of CMHL (prosthesis lateralisation without dislocation from the LPI, fibrosis, malleus ankylosis) prior to prosthesis dislocation, the incidence of prosthesis dislocation over time was estimated using the Kalbfleisch and Prentice method [14]. The Kalbfleish and Prentice method provides an estimate of the cumulative incidence of stapedial prosthesis dislocation where we classify early surgical revision for postoperative SNHL and other cause of CMHL prior to stapedial prosthesis dislocation as competing events. Differences in the cumulative incidence functions among groups were assessed using the Gray’s test [15].

All analyses were performed with statistical programming language R, version 4.0.4 (R Project for Statistical Computing) [16]. The threshold for statistical significance was set to p < 0.05.

## Results

### Population

Eight hundred and fifty-five patients were included for the analysis. The mean age (SD) was 45.3 (11.7) year old (range, 16–82). 529 (61.9%) patients were female. 758 (88.7%) patients had a Teflon piston and 97 (11.3%) Titanium piston. Both groups were similar with respect to age and sex (Table 1). Footplate fenestration was performed with laser in 54.9% of cases, and without (manual footplate perforator only) in 45.1% of cases.

**Table 1:** Characteristics of groups

Variable	Teflon	Titanium	p-value
Number of patients (%)	758 (88.7%)	97 (11.3%)	
Age, mean (SD)	45.2 (11.7)	45.3 (12.3)	1
*Sex*, n (%)			
Males	283 (37.3%)	43 (44.3%)	0.48
Females	475 (62.7%)	54 (55.7%)	
*Footplate fenestration*, n, (%)			
With laser	393 (51.8%)	65 (67.0%)	0.005
Without laser	365 (48.2%)	32 (33.0%)	

Comparisons were performed with Chi^2^ test for proportions comparison, and with a Student’s t-test for quantitative variables

### Primary endpoint

**Fig. 2:**
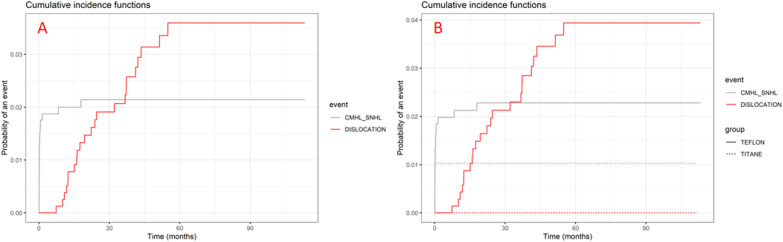
Cumulative incidence functions estimaded by Kalbfleish and Prentice method. CMHL and SNHL prior to dislocation were considered as competing events. All patients (**A**), and comparing Teflon and Titanium pistons (**B**). CMHL_SNHL: other cause of Conductive/Mixed hearing loss and Sensorineural Hearing loss

Median follow-up was 51.7 months (28.4–80.1). Overall, 23 of 855 patients (2.7%) had a prosthesis dislocation. Amongst the 23 dislocation cases, 20 (87.0%) were identified on CT-scan then confirmed intraoperatively and 3 (13.0%) were identified on CT-scan only (revision surgery declined by patients). There was 23 (3.0%) dislocations in the Teflon group, and 0 (0.0%) in the Titanium group. The probability of prosthesis dislocation at two years after surgery was 3.5% in the Teflon group and 0.0% in the Titanium group (Fig. 2). There was no statistical difference in cumulative incidence function between groups (p = 0.12).

### Secondary endpoints

There was 17 (2.2%) revision for other cause of CMHL and SNHL (2 cases of recurrent CMHL with peri-prosthetic fibrosis found intraoperatively, and 15 cases for early SNHL or vertigo) in the Teflon group, and 1 (1.0%) case of early SNHL in the Titanium group.

Amongst the 23 dislocation cases, 3 (13.0%) had severe LPI erosion and malleostapedotomy was performed. Moderate LPI erosion was present in 2 (8.7%) cases and cementoplasty was performed. 16 (69.6%) cases had mild or no incus erosion, allowing conventional incudostapedotomy. 3 (13.0%) patients with dislocation did not undergo revision surgery. Among the 20 cases having undergone revision stapes surgery, 16 (80.0%) were treated with conventional stapes surgery with a titanium prosthesis inserted on the LPI (Big Easy, Medtronic*), 2 (10.0%) with a titanium prosthesis inserted on the LPI stabilized with surgical cement (Otomimix, Olympus*) and 2 (10.0%) with a malleostapedotomy using a nitinol malleus-to-stapes prosthesis (SMart Malleus piston, Olympus*). In 4/23 (17.4%) dislocation cases, a triggering event was identified (2 airplane travel, 1 acute otitis media, and 1 traffic accident).

## Discussion

This is the first study comparing stability of the attachment of the loop onto the LPI for two kinds of manual crimping stapes prostheses, fluoroplastic (Causse, Medtronic) and titanium (Big Easy, Medtronic). Prosthesis dislocation from its attachment to the LPI is the major cause of revision procedures for recurrent CMHL after primary stapedotomy [17–19]. To our knowledge, the incidence of stapes prosthesis dislocation had not been described before.

In this large series of 855 primary stapes procedures over a period of nearly 10 years and with a median follow-up of 51.7 months (28.4–80.1), we found 23 (3.0%) dislocations among the 758 fluoroplastic prostheses and none (0.0%) among the 97 titanium prostheses. The probability of prosthesis dislocation at two years after surgery was 3.5% in the Teflon group and 0.0% in the Titanium group. Although we were unable to show a statistically significant difference between the two groups, these results show a trend toward more stable fixation to the incus over time with titanium.

### Clinical application of the study

Several types or brands of stapes prostheses of different materials are available on the market. Most stapes prostheses require manual crimping, which is crucial for the success of the procedure [20, 21]. The choice of the prosthesis is based on multiple parameters: biocompatibility, safety, hearing results, stability over time, surgeon's habits, price, and, probably increasingly, carbon footprint.

Postoperative hearing results have already been extensively studied, regarding material or surgical technique (partial or total stapedectomy, stapedotomy with manual perforator, microdrill or laser…) [3, 6, 7, 22, 23]. To date, no stapes prosthesis exhibited significant better hearing results over others.

In our daily practice, 0.6 mm diameter fluoroplastic stapes prosthesis is our first choice for primary stapedotomy, whereas 0.5 mm diameter titanium prosthesis is used in selected situations, such as narrow oval window niche, thin or eroded LPI, patient having already been operated on with a titanium prosthesis on the contralateral ear.

### Limits

There are several limitations to our study: retrospective design, risk of loss of follow-up of patients with prosthesis dislocation managed in other center, and routine use of fluoroplastic prostheses versus selected use of titanium prostheses. The risk of loss of follow-up cannot be excluded, but it is in the author’s opinion that it is unlikely to represent a significant number of patients, given that the study was performed at a referral center for stapes surgery and revision stapes surgery. There might be a selection bias on the choice of prosthesis, because the titanium prostheses were implanted in selected cases. However, this is not a favorable bias for the statistical results and could rather support our results, since the selected cases may have had a higher risk of prosthesis dislocation.

There was a difference in the use of laser for footplate fenestration between Teflon and Titanium groups, but it is not in the author’s opinion that it would impact stability of piston attachment to the LPI. Manual perforator was routinely used till 2015, then diode fiber laser was most regularly used, except in cases where laser was not appropriate (mainly, dehiscent facial nerve) or for academic concerns (residents’ teaching). We also have been using increasingly more Titanium prostheses, leading to this difference. In the opinion of the authors, this would not have been a bias because the laser and non-laser fenestrations are calibrated with the same manual perforator and it would not have changed the stability of the shaft of the prosthesis.

### Comparison with other studies

To our knowledge, there is no study comparing stability of prosthesis stability over time regarding the material used. Our study does not address hearing outcomes with regard to material used, since this has been thoroughly described in other studies [6, 24]. We observed 20% moderate and severe incus erosion in our study. In comparison, Lundman et al*.*’s study had 35% erosion in 227 first revision cases, Blijleven et al. 5% erosion in 63 cases, and Lippy et al. 23.5% erosion in 522 revisions [17–19]. The variability of those results could be explained by the lack of clear definition of incus erosion in revision stapedotomy. However, status of the LPI is crucial for deciding the most appropriate surgical technique in revision: standard technique in cases with no or mild erosion, standard technique with surgical cement stabilization in case with moderate erosion, malleostapedotomy in case with severe erosion [25].

Lundman et al. [17] reported a longer median delay from primary to revision surgery (7 years 6 months; min = 3 months; max = 50 years) but our results are not directly comparable since revision patients that had undergone surgery before 2013 were excluded from our study. In our series a triggering event causing the dislocation was found in only 17% of the cases (4/23). In comparison, Puxeddu et al. [26] described 2/44 (4.5%) triggering traumatic events.

## Conclusion

In this series of 855 primary stapes surgeries comparing the risk of dislocation from the incus of two popular manual crimped prosthesis, we have observed 3% of dislocation of Teflon prosthesis, against 0% of dislocation of titanium prosthesis. This is suggestive of a better stability of titanium prosthesis over time, although we failed to establish statistical significance. Further prospective randomized studies could be valuable to assess our findings.

## Data Availability

The dataset supporting the conclusions of this article is available in the OPENICPSR repository at https://doi.org/10.3886/E181742V2.

## Abbreviations

CMHL: Conductive or mixed hearing-loss
LPI: Long process of incus
CT: Computed tomography
SNHL: Sensorineural hearing loss
